# Absorption-Driven Near-Field EMI Shielding of Si-CNT Composite for LED Displays: A Solution for the Transition from Reflective Inefficiency to Absorptive Suppression

**DOI:** 10.3390/ma19163548

**Published:** 2026-08-21

**Authors:** Young-Soon Kim, Sun-Ho Choi, Sumin Jung, Jaeun Jin, Minjin Oh, Suk-Dae Lim, Hong-Gun Kim

**Affiliations:** 1 Institute of Carbon Technology, Jeonju University, Jeonju 55069, Republic of Korea; kyscjb@jj.ac.kr; 2 Department of Carbon Convergence Engineering, Jeonju University, Jeonju 55069, Republic of Korea; rokmwinning2014@jj.ac.kr; 3 Department of Carbon Convergence and Energy Engineering, Jeonju University, Jeonju 55069, Republic of Korea; sumin5681@jj.ac.kr; 4 L&D TECH Co., Ltd., Gunsan 54078, Republic of Korea; mountain-man@hanmail.net (J.J.); omj6906@naver.com (M.O.); 5 The DEUM Co., Ltd., Iksan 54588, Republic of Korea; powersd@hanmail.net

**Keywords:** electromagnetic interference (EMI) shielding, composite materials, near-field noise suppression, absorption-dominant mechanism, silicone-carbon nanotube (Si-CNT) composite, LED display application, recycled carbon fiber nonwoven

## Abstract

The issue of near-field electromagnetic interference (EMI) is being made worse by the widespread use of highly integrated electronic devices, including commercial LED displays. Although highly conductive pristine carbon networks, like recycled carbon fiber nonwovens (rCFNWs), have excellent far-field shielding effects (~43 dB) in theory, their purely reflection-oriented mechanisms cause severe secondary signal interference in practical near-field applications due to reflective inefficiency. This study suggests employing a custom-formulated silicone-carbon nanotube (Si-CNT) composite to switch to an absorption-based shielding mechanism in order to get around these restrictions. This study used FE-SEM, Raman spectroscopy, XPS, ICP-AES, FTIR, and TGA-DTG to systematically investigate the morphological, chemical, and thermal properties of the rCFNW, Si-CNT composite, and a Cu-integrated variant (Si-CNT-Cu). Surface reflection was greatly reduced by adding CNTs to the silicone matrix, converting the materials into absorption-oriented localized shielding composite materials (~16 dB). Both the designed Si-CNT membrane and the Cu-integrated Si-CNT-Cu product totally eliminated the 850 MHz switching noise peak (>40 dBuV) in real near-field tests of commercial LED modules running under worst-case conditions (5.36 A). Additionally, the bare Si-CNT membrane showed a lower coefficient of thermal expansion (CTE) in the thermomechanical analysis (TMA) than the Si-CNT-Cu product. On the other hand, the macroscopic integration of Cu wires in the Si-CNT-Cu composite provided remarkable thermomechanical stability, preventing thermal softening by preserving an exceptionally high storage modulus of 97.54 MPa at 198 °C, according to dynamic mechanical analysis (DMA). These findings show that using absorptive suppression to overcome near-field inefficiency is a very successful method for creating dependable EMI shielding composite materials in high-power electronic systems.

## 1. Introduction

The electromagnetic environment is becoming more complex due to the quick development of contemporary electronic information technologies and highly interconnected communication systems [1,2,3]. Specifically, the power density, switching rates, and component downsizing of commercial light-emitting diode (LED) display modules are reaching previously unheard-of levels [4,5]. Although these developments offer faster data transmission and better visual performance, they also cause significant electromagnetic interference (EMI) because of the high working currents and fast-switching clock signals in the dense driving circuit [6,7]. Additionally, the electromagnetic compatibility (EMC) of these high-power LED light sources is a crucial issue influencing their overall performance and regulatory compliance, according to current market surveillance and evaluation studies [8]. This strong near-field electromagnetic radiation can lead to serious signal crosstalk, logic mistakes, and gradual deterioration of nearby internal components in highly integrated electronic packages, ultimately jeopardizing the system’s overall operational stability [9,10,11]. Thus, it is now urgently necessary to create localized EMI shielding materials that can efficiently suppress near-field electromagnetic noise without interfering with the correct operation of precision electronic equipment [12,13,14,15].

Highly conductive carbon-based fibrous materials have historically been thoroughly studied as strong, lightweight options for EMI shielding [16,17]. Among them, recycled fibrous structures and carbon fiber nonwoven (CFNW) fabrics offer exceptional benefits such as a high aspect ratio, superior chemical resistance, and a highly linked three-dimensional (3D) conductive network [18,19]. Recycled carbon fiber nonwovens (rCFNWs) have attracted a lot of attention lately due to their excellent structural integrity and highly linked web designs, in addition to being a sustainable and environmentally friendly option [20]. Additionally, it has been demonstrated that incorporating highly conductive metals, such as copper, into carbon fiber nonwoven fabrics is a remarkable method for attaining ultrahigh EMI shielding performance while retaining lightweight and flexible qualities [21,22,23]. Because of their unusually high electrical conductivity, several independent research groups assessing the EMI shielding qualities of pure carbon fiber networks have shown remarkable total shielding efficiency (SE_T_) in far-field studies [24,25]. However, because of the significant impedance mismatch between the highly conductive carbon surface and the open space, the shielding mechanism of these bare carbon networks is primarily controlled by surface reflection (SE_R_) rather than absorption (SE_A_) [25,26,27]. When used as a near-field shield directly over high-frequency integrated circuits (ICs), this reflection-dominant behavior causes severe “reflective inefficiency”. Instead of dissipating, the incoming electromagnetic waves bounce back toward the source of the noise, causing secondary signal interference and permitting electromagnetic leakage via minuscule physical gaps [28]. Therefore, a paradigm shift from reflective shielding to absorptive suppression is required to accomplish realistic near-field noise reduction.

Integrating a flexible and electrically insulating polymer matrix is thought to be a highly successful strategy to overcome the drawbacks of highly reflecting pure carbon networks [29,30]. A popular polysiloxane elastomer, silicone rubber (SR), has exceptional thermomechanical flexibility, remarkable high-temperature stability, and built-in electrical insulation [31,32]. The non-conductive silicone rubber functions as an ideal impedance-matching dielectric layer at the material interface when used as a matrix for EMI shielding composites [33]. This creates a crucial basis for absorption-dominant shielding methods by enabling incident electromagnetic waves to pass through the shielding membrane’s surface rather than being instantly reflected [34,35].

However, in order to create an internal wave-dissipating network, secondary conductive nanofillers must be added because pure silicone rubber is completely transparent to electromagnetic waves [36]. For this aim, carbon nanotubes (CNTs) and graphene—dimensional (1D/2D) conductive nanomaterials with exceptional electron mobility and an ultra-high aspect ratio—are attractive options [37,38,39]. Even at low filler loadings, the addition of CNTs to the silicone rubber matrix promotes the development of a strong, percolated conductive network [40]. Thick dielectric polymer layers surround the conductive carbon fibers and carbon nanotubes in a tailored silicone rubber/CNT (Si-CNT) composite, avoiding instantaneous surface reflection. Simultaneously, the incident microwaves trapped within the composite undergo multiple internal reflections and interfacial scattering among the dense CNT networks, ultimately dissipating the electromagnetic energy as heat via ohmic losses and polarization relaxation. This synergistic effect perfectly converts the composite into a highly efficient, absorption-driven shielding material suitable for localized near-field applications.

Despite extensive research on carbon-based polymer composites for EMI shielding, a persistent challenge lies in balancing high absorption efficiency with seamless device-level integration for practical electronics. To bridge this critical gap, a high-performance custom-formulated Si-CNT composite and its Cu-integrated variant (Si-CNT-Cu) were systematically developed and evaluated in this study as localized EMI shielding materials for commercial LED displays. Our goal is to address the fundamental disparity between the severe reflecting inefficiency of highly conductive bare carbon networks (recycled CFNW) in near-field situations and their predicted far-field shielding performance, successfully achieving an absorption-dominant shielding mechanism within an optimized 2 mm thickness. The morphological, chemical, and thermal properties of the fabricated composites were comprehensively characterized utilizing FE-SEM, Raman spectroscopy, XPS, ICP-AES, FTIR, and TGA-DTG. Furthermore, the dimensional stability and viscoelastic thermomechanical behaviors were extensively evaluated through TMA and DMA to guarantee high-temperature operational reliability. Ultimately, unlike conventional studies restricted to far-field coaxial evaluations, the novelty of this work is underscored by its comprehensive demonstration of real-world near-field noise suppression. Through rigorous practical near-field EMI mapping of an active LED module operating under maximum current (5.36 A)—validated under standardized protocols at an official testing institute (Jeonbuk Institute of Automotive Convergence Technology, JIAT)—we unequivocally demonstrate the successful paradigm shift toward an absorption-dominant shielding mechanism, proving the exceptional capability of the Si-CNT shielding materials in eradicating severe high-frequency switching noise.

## 2. Materials and Methods

### 2.1. Materials

The recycled carbon fiber nonwoven (rCFNW) was supplied by Hansol Chemical, Inc. (Iksan, Republic of Korea). The silicone-carbon nanotube (Si-CNT) composite was custom-formulated and provided by CNT Solution Co., Ltd. (Cheonan, Republic of Korea), wherein the CNT concentration was specifically controlled at 3 wt% within the silicone matrix to optimize the electromagnetic absorption characteristics. Due to the proprietary nature of the specialized manufacturing process by the supplier, the specific details regarding dispersion methods and secondary chemical treatments remain confidential; however, the composite exhibited a well-established uniform network structure as confirmed by structural characterizations. The copper wire-integrated Si-CNT composite (Si-CNT-Cu) was obtained from Biolamp Inc. (Jeonju, Republic of Korea) as a commercial electromagnetic shielding product to evaluate its practical near-field noise suppression capabilities and thermomechanical stability. Specifically, the embedded copper component consists of a plain-woven copper mesh made of solid circular wires with a precise diameter of approximately 100 μm. This mesh is structured as a uniform planar layer oriented parallel to the membrane surface within the matrix. Additionally, the commercial LED module, which was utilized as the electromagnetic noise source for the practical EMI shielding application tests, was manufactured and supplied by L&D TECH Co., Ltd. (Gunsan, Republic of Korea).

### 2.2. Practical Near-Field Setup and Interfacial Insulation

To systematically evaluate the practical near-field EMI shielding effectiveness, a commercial LED module was utilized as the active noise source. For the measurement setup, two distinct inspection zones were defined on the rear driving circuitry. A broad baseline scanning area was established to measure the intrinsic radiated emission of the bare module. Subsequently, a specific localized target area was mapped over the high-frequency driving ICs, where the precisely cut Si-CNT and Si-CNT-Cu membranes were directly placed for noise suppression tests. To prevent operational DC short circuits between the active digital components and the partially conductive carbon networks within the composite, the LED module supplied by L&D TECH Co., Ltd. was pre-treated by the manufacturer with a liquid conformal insulating coating over the exposed PCB.

### 2.3. Characterization

The surface morphologies and structural characteristics of the samples were examined using a field-emission scanning electron microscope (FE-SEM, SU8600, Hitachi, Tokyo, Japan). The structural crystallinity and the presence of defects in the carbonaceous materials were evaluated using a Raman spectrometer (HR-800, Horiba LabRAM, Kyoto, Japan). The Raman spectra were recorded utilizing a 532 nm laser excitation source, a grating resolution of 1800 lines/mm, an acquisition time of 5 s, an accumulation count of 5, and a 100% neutral density filter over a spectral range of 100 to 4000 cm^−1^. The chemical states and surface elemental compositions were analyzed utilizing X-ray photoelectron spectroscopy (XPS, K-Alpha, Thermo Fisher Scientific, Waltham, MA, USA). Additionally, the bulk concentrations of carbon, oxygen, silicon, and other trace elements were precisely detected using an inductively coupled plasma atomic emission spectrometer (ICP-AES, Optima 7300 DV, Perkin Elmer, Waltham, MA, USA). The surface functional groups and chemical bonding states were identified via Fourier transform infrared (FTIR) spectroscopy (Nicolet iS5, Thermo Fisher Scientific, Waltham, MA, USA). The thermal stabilities and degradation behaviors were investigated using a thermogravimetric analyzer (Mettler-Toledo, TGA/DSC1, Columbus, OH, USA) from room temperature to 1000 °C under a nitrogen atmosphere. All of the aforementioned morphological and chemical characterizations (FE-SEM, Raman spectroscopy, XPS, ICP-AES, FTIR, and TGA-DTG) were performed at the Center for Research Facilities, Chungnam National University (Daejeon, Republic of Korea).

The macroscopic thermomechanical properties, including the coefficient of thermal expansion (CTE), were measured using a thermomechanical analyzer (TMA, Q400, TA Instruments, New Castle, DE, USA). The viscoelastic properties, such as storage modulus, loss modulus, and tan delta, were characterized using a dynamic mechanical analyzer (DMA, Q800, Waters Corporation, Milford, MA, USA) over a temperature range from −100 °C to 300 °C. These thermomechanical properties (TMA and DMA) were analyzed at the Center for University-Wide Research Facilities, Jeonbuk National University (Jeonju, Republic of Korea).

Finally, the intrinsic EMI shielding effectiveness of the composites, including the comprehensive scattering parameters (S_11_, S_21_, S_12_, and S_22_) necessary for decoupling the reflection and absorption mechanisms, was evaluated in the frequency range of 0.4 to 1.6 GHz. The measurements were conducted using a vector network analyzer (VNA, E5080A, Keysight Technologies, Santa Rosa, CA, USA) connected to an ASTM D4935-18 [41] compliant coaxial transmission line specimen holder equipped at Jeonju University (Jeonju, Republic of Korea). To ensure the statistical robustness and reliability of the EMI shielding evaluation, all specific measurements were performed independently at least three times for each sample configuration. Due to the large volume of sample formulations evaluated and to prevent the severe compression variations (thickness distortion) typical when clamping thick elastomers, a modified non-destructive screening approach was utilized for the VNA measurements. Large-area composite sheets (20 cm × 30 cm) were placed directly over the flanged aperture. While this unbolted setup may introduce minor edge-effect leakage compared to the strict ASTM standard, it effectively preserved the pristine 2 mm thickness of the flexible membranes. This provided highly reliable comparative data to observe the relative transition from reflection- to absorption-dominant shielding mechanisms. The definitive practical shielding performance was subsequently validated through the near-field LED module evaluation, the precise structural setup of which is detailed in Section 2.2 and schematically illustrated in Section 3.11.

For the practical application validation, the spatial and spectral radiated electromagnetic emissions from the LED module were rigorously validated under standardized measurement protocols at the Jeonbuk Institute of Automotive Convergence Technology (JIAT, Gunsan, Republic of Korea). The 2D and 3D spatial EMI mappings were acquired using an automated near-field EMI Scanner (RCE-40H, ERETEC INC., Gunpo, Republic of Korea). Specifically, the near-field scanning was performed in a non-contact manner at a constant height of 1 mm above the target surface, utilizing a precise spatial resolution of 1 mm along both the horizontal and vertical axes. To strictly decouple the intrinsic electromagnetic shielding effect from geometric distance attenuation, the automated scanner was programmed with a fixed absolute *Z*-axis coordinate. The vertical distance between the magnetic field probe and the underlying PCB surface was strictly locked across all measurements, ensuring that the physical distance from the noise source remained absolutely identical for both the bare baseline and the shielded conditions. The quantitative far-field radiated emission spectra were measured in a 10 m semi-anechoic chamber equipped with an EMI test receiver (ESR 7, Rohde & Schwarz, Munich, Germany) and a broadband hybrid antenna (HLP-2006C, TDK, Tokyo, Japan) following the CISPR 25 testing procedures.

## 3. Results and Discussion

### 3.1. Surface Morphologies

**Figure 1:**
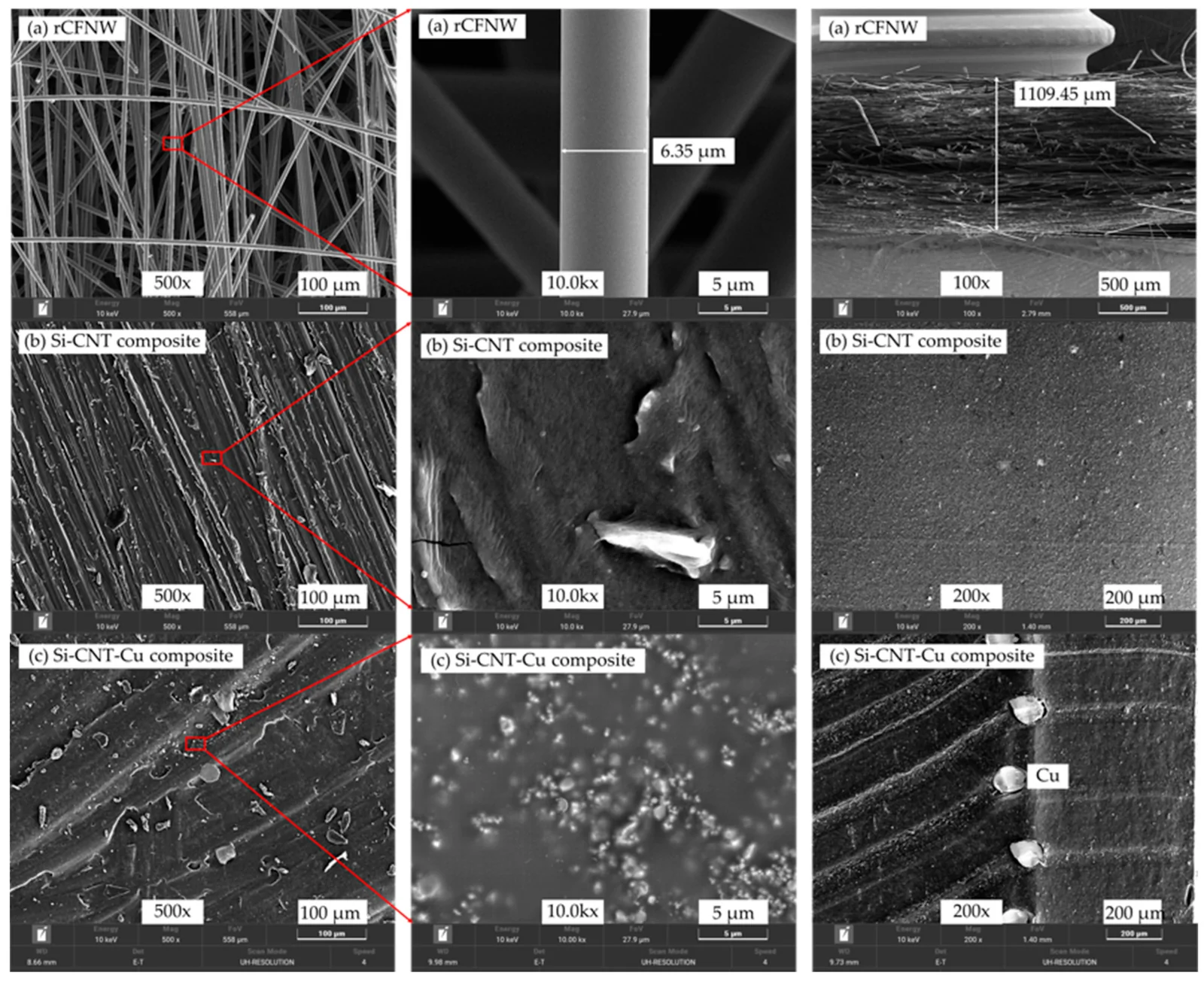
FE-SEM micrographs showing the surface and cross-sectional morphologies of the (**a**) recycled carbon fiber nonwoven (rCFNW), (**b**) Si-CNT composite, and (**c**) Si-CNT-Cu composite.

Figure 1 presents the FE-SEM micrographs detailing the surface and cross-sectional morphologies of (a) the recycled carbon fiber nonwoven (rCFNW), (b) the custom-formulated Si-CNT composite, and (c) the Cu-integrated Si-CNT-Cu composite. The pristine rCFNW exhibited a randomly entangled fibrous network; the enlarged surface image confirmed the presence of carbon fibers with an average diameter of approximately 6.35 μm. Its cross-section revealed a highly porous nonwoven architecture with a measured thickness of about 1.11 mm.

In stark contrast to the highly porous framework of the rCFNW, both the Si-CNT and Si-CNT-Cu composites demonstrated dense, thoroughly impregnated structures where the silicone matrix completely encapsulated the carbonaceous fillers. Upon closer comparison, the surface of the bare Si-CNT composite featured deep grooves with relatively large agglomerated particles scattered between the striations. On the other hand, the Si-CNT-Cu composite exhibited smoother surface striations and a higher, more uniform density of fine particles. Furthermore, its cross-sectional view clearly revealed the macroscopic Cu wire firmly embedded within the dense polymer matrix. While the full cross-sectional thickness of the highly porous rCFNW (~1.11 mm) was entirely visible at 100× magnification, the higher magnification (200×) utilized for observing the internal microstructures of the Si-CNT and Si-CNT-Cu composites inherently restricts the field of view, preventing the entire cross-section from being captured in a single micrograph. Nevertheless, macroscopic physical measurements confirmed that the total thickness of the actual Si-CNT and Si-CNT-Cu samples used for the characterizations in this study was uniformly controlled to 2 mm.

### 3.2. Raman Spectroscopy

**Figure 2:**
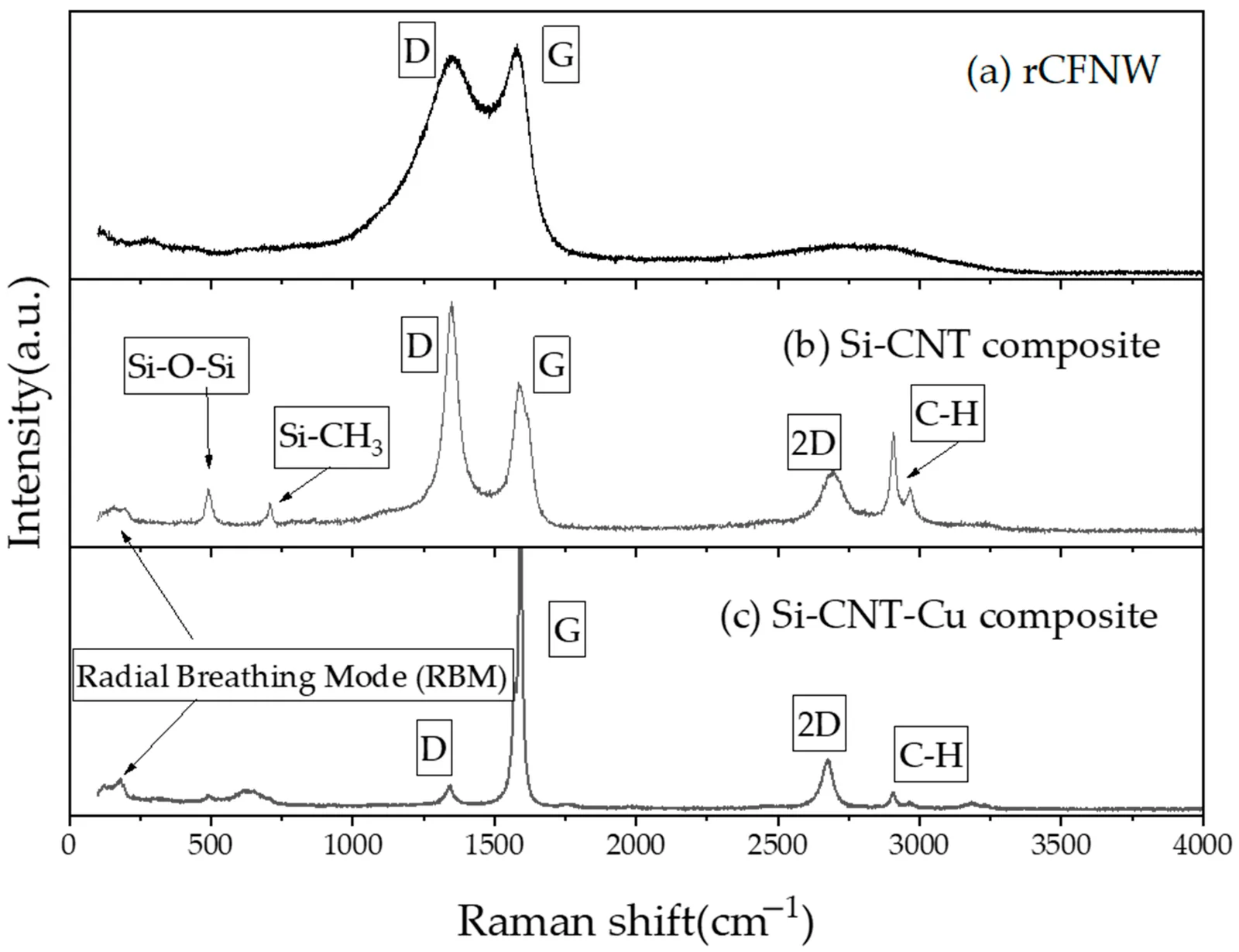
Raman spectroscopy of the (**a**) recycled carbon fiber nonwoven (rCFNW), (**b**) Si-CNT composite, and (**c**) Si-CNT-Cu composite.

Figure 2 presents the Raman spectra of the (a) rCFNW, (b) Si-CNT composite, and (c) Si-CNT-Cu composite, recorded over a range of 100 to 4000 cm^−1^. The characteristic vibrational modes corresponding to the graphitic carbon structures, silicone rubber matrix, and carbon nanotubes are clearly distinguished.

When comparing the pristine rCFNW in Figure 2a with the composites, distinct spectral evolutions were observed. In all samples, the D-band (disorder) was observed at approximately 1350 cm^−1^, originating from structural defects, amorphous carbon phases, or edge configurations within the *sp^2^* hybridized graphitic lattice. In the pristine rCFNW spectrum, this D-band was exceptionally broad and intense, reflecting a high density of inherent structural defects resulting from the recycling process. The G-band (graphitic), located around 1580 cm^−1^, corresponds to the primary in-plane stretching mode (*E_2g_* phonon mode) common to all *sp*^2^ carbon materials. The relative intensity ratio of the D-band to the G-band (*I_D_/I_G_*) serves as a key indicator of the structural crystallinity. Additionally, the symmetric 2D-band appearing near 2700 cm^−1^ is attributed to two-phonon lattice vibrations and is widely utilized to evaluate the layer thickness and electronic properties of graphene-like sheets or CNT walls.

A direct comparison revealed the successful integration of the hybrid components. Detected exclusively in the low-frequency region of 100 to 300 cm^−1^ is the radial breathing mode (RBM) in Figure 2b,c. The distinct emergence of this RBM in both the Si-CNT and Si-CNT-Cu composites, which was entirely absent in the bare rCFNW, explicitly confirms the retention of single-walled carbon nanotubes (SWCNTs) within the blended matrix, with its frequency being inversely proportional to the tube diameter. Furthermore, the transition from a bare carbon network to a polymer composite is corroborated by the distinct chemical signatures of the silicone rubber matrix (PDMS-related modes) uniquely appearing in the spectra of (b) and (c). The peak at approximately 2900 cm^−1^ corresponds to the C-H stretching vibrations. Characteristic backbone vibrations were also exclusively identified at lower wavenumbers in the composites, including the Si-O-Si symmetric stretching mode near 490 cm^−1^ and the Si-CH_3_ stretching mode around 705 cm^−1^.

Finally, a comparison between the Si-CNT and Si-CNT-Cu composites revealed that their Raman spectra were virtually identical. This crucial observation indicates that the macroscopic physical integration of the Cu wire does not alter or disrupt the fundamental chemical bonding and *sp*^2^ carbon framework of the surrounding Si-CNT matrix.

### 3.3. X-Ray Photoelectron Spectroscopy

**Figure 3:**
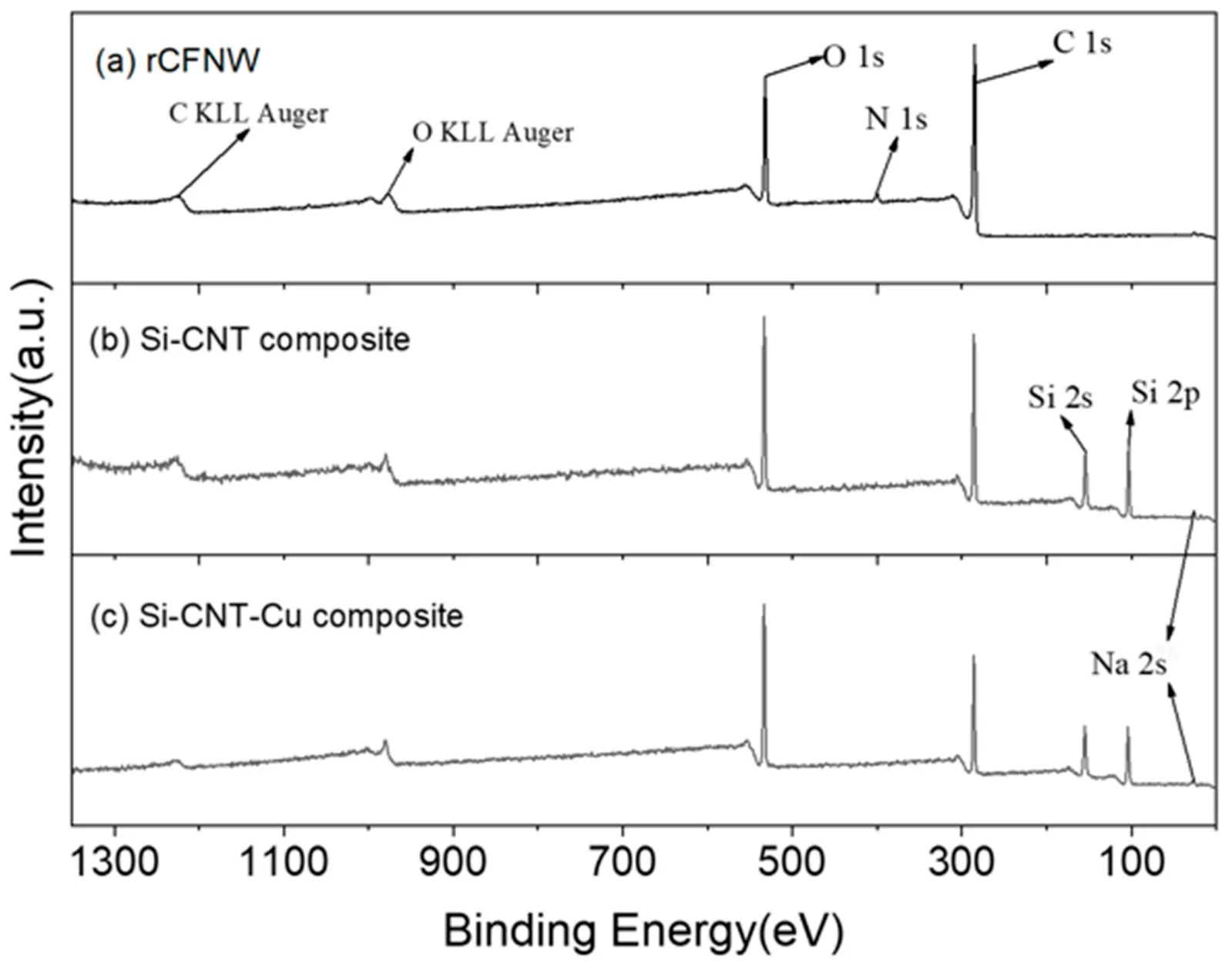
XPS wide-scan survey spectra of the (**a**) rCFNW, (**b**) Si-CNT composite, and (**c**) Si-CNT-Cu composite.

Figure 3 illustrates the XPS wide-scan survey spectra of the (a) rCFNW, (b) Si-CNT composite, and (c) Si-CNT-Cu composite over a binding energy range of 0 to 1350 eV. The spectra provide definitive evidence regarding the elemental composition and surface chemical states of each sample, clearly showing the transition from a bare carbon network to a silicone-encapsulated composite. In Figure 3a, the survey spectrum of the pristine recycled carbon fiber nonwoven (rCFNW) predominantly displayed a sharp carbon peak (C 1s) at approximately 284.8 eV and an oxygen peak (O 1s) at around 532 eV. These peaks represent the core graphitic framework and surface oxygen-containing functional groups inherited from the recycling or sizing treatments. A minor nitrogen peak (N 1s) was also visible near 400 eV, and characteristic C KLL and O KLL Auger transitions were well-resolved at higher binding energies. Following the integration of the carbonaceous fillers into the silicone rubber matrix, Figure 3b reveals a dramatic change in the surface chemical composition. Distinctive silicon peaks emerged dominantly in the lower binding energy region. Specifically, the Si 2s and Si 2p core-level peaks were clearly observed at approximately 154 eV and 102 eV, respectively, confirming the successful, uniform encapsulation of the carbon network by the siloxane Si-O-Si backbone structures. Finally, the spectrum of the hybrid ternary composite (Si-CNT-Cu) in Figure 3c virtually mirrored that of the Si-CNT composite, retaining all of the characteristic peaks of both the carbonaceous network and the robust silicone matrix without any significant peak shifts. This indicates that the macroscopic integration of the Cu wire does not induce unwanted chemical degradation on the composite surface. Notably, a trace emission peak corresponding to Na 2s was detected at a very low binding energy of 26 eV, which likely stems from residual impurities or processing agents introduced during fabrication.

### 3.4. Elemental Analysis

As summarized in Table 1, the bulk elemental composition evaluated via inductively coupled plasma-atomic emission spectroscopy (ICP-AES) revealed distinct changes in the atomic percentages across samples, perfectly correlating with the surface chemical state changes observed in the XPS results. The pristine rCFNW predominantly consisted of carbon (91.7 at%) with minor amounts of nitrogen (5.74 at%) and oxygen (2.56 at%), verifying its high-purity carbonaceous nature. Upon the incorporation of the silicone matrix and carbon nanotubes, the Si-CNT composite exhibited a significant reduction in the relative carbon content (43.81 at%). This is directly accompanied by the prominent emergence of silicon (20.3 at%) and a substantial increase in oxygen (35.69 at%), which strictly reflects the elemental makeup of the siloxane (Si-O-Si) polymer matrix encapsulating the conductive fillers. Following the integration of the copper reinforcement, the Si-CNT-Cu composite displayed a highly stable base matrix composition with only slight variations in the bulk C, O, and Si contents. Most notably, an increase in other unclassified trace elements (3.79 at%) was detected by the highly sensitive ICP-AES measurement, further indicating the successful macroscopic integration of the Cu component into the hybrid structure. Ultimately, these highly consistent compositional trends between the bulk ICP-AES and surface XPS analyses strongly corroborate the successful formation and chemical stability of the engineered composite materials.

### 3.5. Fourier Transform Infrared (FTIR) Spectroscopic Analysis

**Figure 4:**
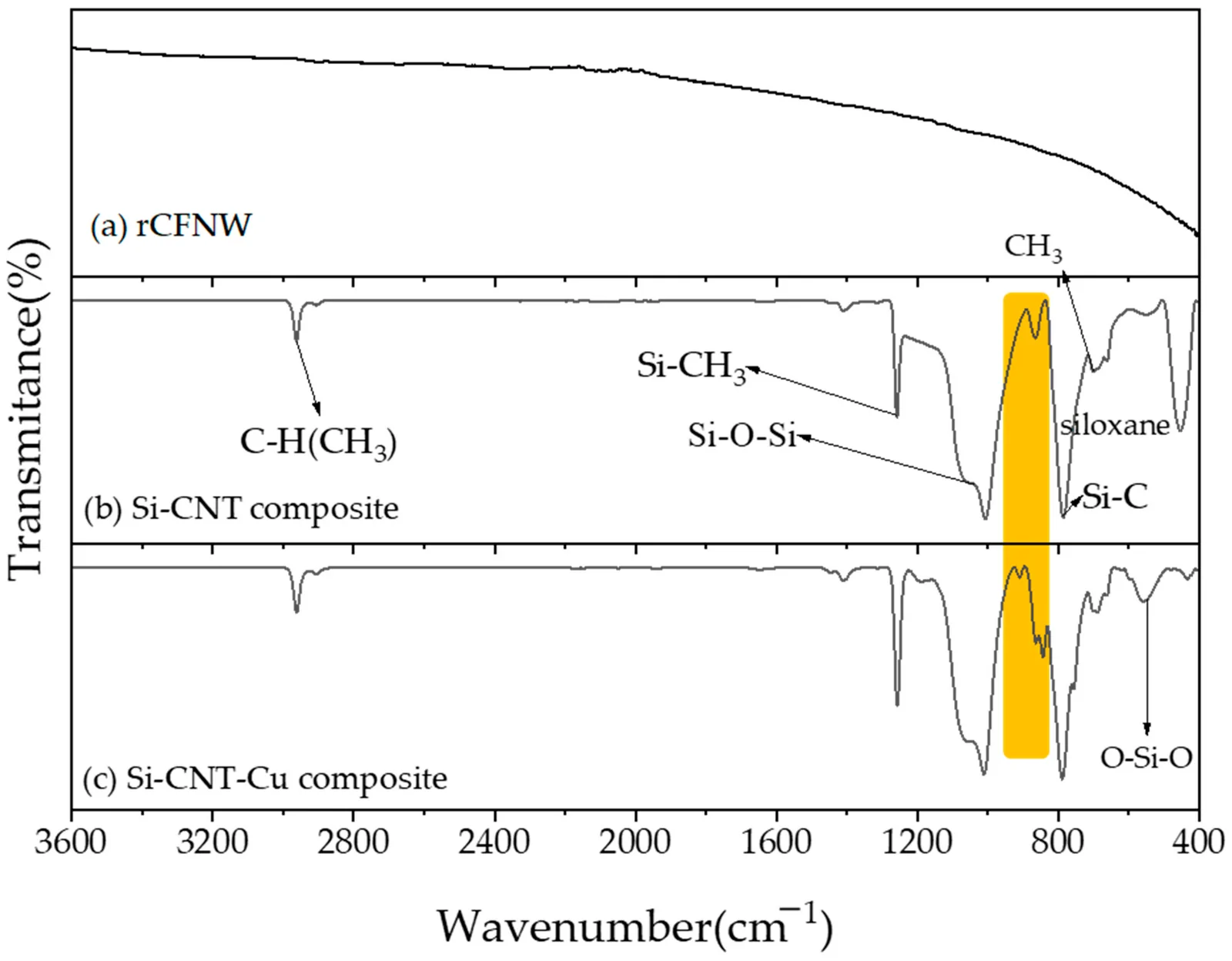
FTIR curves of the (**a**) rCFNW, (**b**) Si-CNT composite, and (**c**) Si-CNT-Cu composite.

The chemical structures and functional groups of the materials were systematically investigated using Fourier transform infrared (FTIR) spectroscopy, as presented in Figure 4. The spectrum of pristine rCFNW (Figure 4a) displayed a featureless profile with a flat baseline and no discernible absorption bands. This spectral signature is a typical characteristic of highly carbonized, chemically inert carbonaceous structures, confirming the absence of significant surface functional groups. In stark contrast, the FTIR spectra of both the Si-CNT (Figure 4b) and Si-CNT-Cu (Figure 4c) composites exhibited strong and well-defined characteristic bands originating from the silicone rubber matrix. The prominent sharp peak at 2962.29 cm^−1^ is assigned to the asymmetric C-H stretching of methyl (-CH_3_) groups, while the sharp band at 1259.36 cm^−1^ corresponds to the symmetric bending vibration of Si-CH_3_ groups. The successful formation of the hybrid organic–inorganic network was clearly demonstrated by the intense and broad absorption band centered at 1037.57 cm^−1^, which represents the asymmetric stretching vibration of the siloxane (Si-O-Si) backbone. Furthermore, the complex multi-modal region near 800 cm^−1^ revealed overlapping vibration modes, including Si-C stretching at 788.78 cm^−1^ and CH_3_ rocking at 700.07 cm^−1^, which serve as fingerprint bands for the silicone structure. The distinct absorption peak resolved at the lower wavenumber of 457.07 cm^−1^ is explicitly assigned to the O-Si-O bending vibration of the siloxane network.

Upon the integration of the core copper (Cu) wire into the Si-CNT matrix (Figure 4c), the primary characteristic bands of the siloxane backbone remained largely intact, indicating that the bulk organic–inorganic framework is structurally preserved. However, a careful comparison revealed a distinct spectral difference in the highlighted yellow region (approximately 900–950 cm^−1^). In the Si-CNT-Cu composite, this specific absorption band became noticeably more pronounced and deeper compared to the bare Si-CNT composite. This subtle yet clear alteration suggests the presence of local interfacial interactions between the surrounding matrix and the surface of the encapsulated Cu wire. The increased intensity in this specific wavenumber range is likely attributed to localized changes in the siloxane network conformation near the metal surface, the interaction of residual hydroxyl (-OH) groups, or the potential formation of weak interfacial bonds (such as Si-O-Cu or C-O-Cu) at the boundary. Nevertheless, because the main skeletal bands (e.g., Si-O-Si at 1037.57 cm^−1^ and O-Si-O at 457.07 cm^−1^) did not undergo significant shifts or degradation, it can be concluded that these specific interactions were strictly confined to the interface. This interfacial bonding effectively secures the Cu wire without compromising the overall structural integrity of the hybrid composite. Although the specific details of any proprietary surface treatments applied to the commercial Cu wire prior to encapsulation are undisclosed, these observed spectral enhancements confirm the presence of localized interfacial bonding (e.g., Si-O-Cu). These bonds play a crucial role in securing the copper mesh within the silicone matrix, providing baseline interfacial stability against micro-cracking prior to extreme thermomechanical stress.

### 3.6. Thermal Behavior

**Figure 5:**
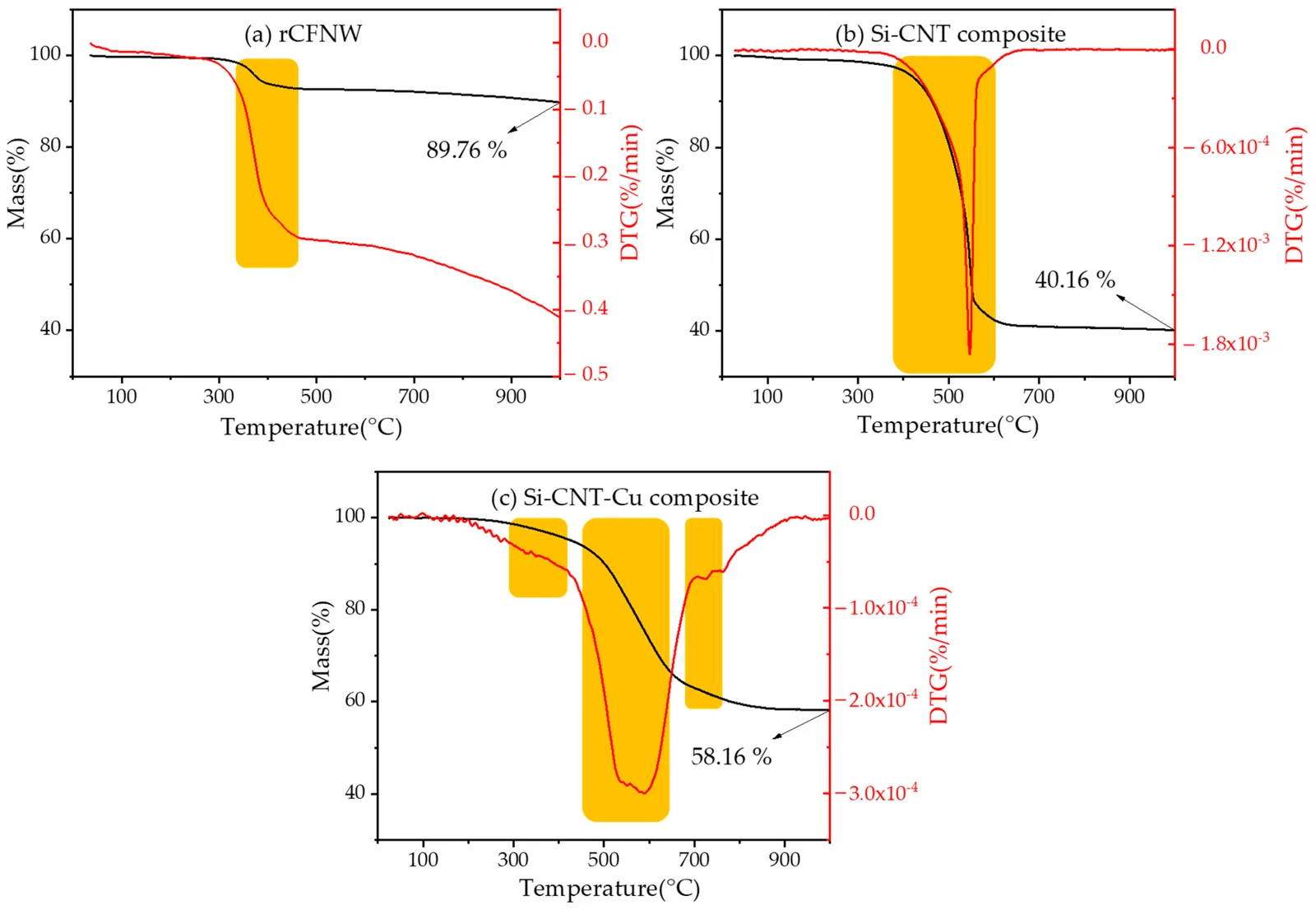
TGA and DTG curves of the (**a**) rCFNW, (**b**) Si-CNT composite, and (**c**) Si-CNT-Cu composite.

The compositional differences among the samples are clearly reflected in their distinct degradation profiles, which perfectly correlate with the previous surface and bulk chemical analyses. As shown in Figure 5a, the pristine rCFNW exhibited excellent thermal stability, featuring a minor weight loss step of 9.24% with a corresponding DTG peak at ~400 °C. It retained a high residual mass of 89.76% at 1000 °C. This highly stable thermal profile perfectly aligns with the XPS and ICP-AES results, which confirmed a high-purity carbonaceous framework (91.7 at% carbon). The minor initial weight loss can be primarily attributed to the thermal decomposition of the surface oxygen-containing functional groups (identified by the O 1s peak in XPS in Figure 3a) or the volatilization of residual sizing agents from the recycling process. In contrast, the TGA curve for the Si-CNT composite (Figure 5b) revealed a much larger weight loss step of approximately 59.84%, accompanied by a prominent DTG peak at ~550 °C. This massive degradation step corresponds directly to the thermal decomposition of the siloxane (Si-O-Si) backbone and organic methyl groups of the silicone rubber. This behavior is strongly supported by the ICP-AES data (Table 1), which demonstrated a dramatic compositional shift—specifically, a significant reduction in carbon content paired with the prominent emergence of Si (20.3 at%) and O (35.69 at%)—confirming the heavy presence of the combustible polymer matrix. This decomposition left a residual mass of 40.16%, which consisted of the thermally stable CNTs, carbon fibers, and inorganic silica ash. Following the addition of the copper reinforcement, the Si-CNT-Cu composite (Figure 5c) displayed a more complex degradation behavior, evidenced by multiple distinct DTG peaks at ~300 °C, ~600 °C, and 800 °C. The overall weight loss step was reduced to approximately 41.84%, resulting in a mathematically increased final residue of 58.16%. This elevated residual mass is a direct macroscopic reflection of the ICP-AES analysis (Table 1), which detected an increase in heavy, unclassified elements (3.79 at%) representing the Cu component. Since the robust macroscopic copper wire acts as a highly stable inorganic metallic filler that does not volatilize at these temperatures, it naturally elevates the overall residual mass. Furthermore, the multi-step degradation profile suggests a complex interfacial thermal dynamic, potentially influenced by the excellent thermal conductivity of the Cu core accelerating internal heat transfer, or its localized catalytic interaction with the surrounding silicone matrix during thermal decomposition.

### 3.7. Thermomechanical Analysis (TMA)

**Figure 6:**
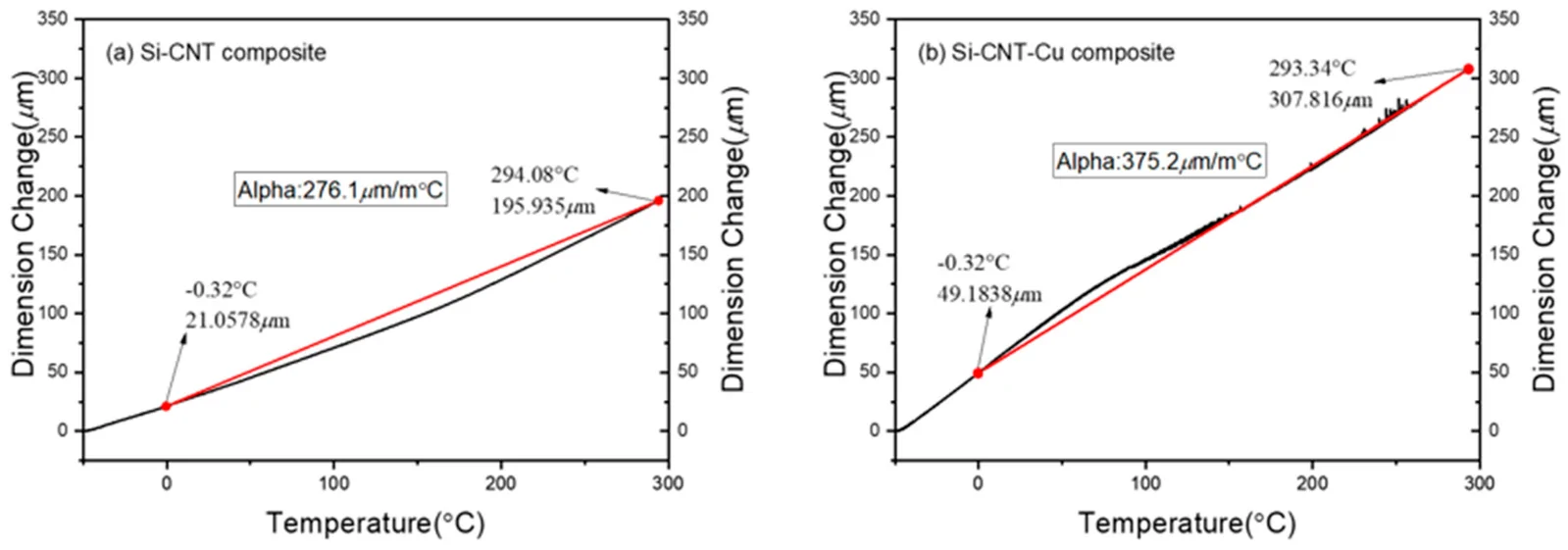
Thermomechanical analysis (TMA) curves showing the dimensional change and the coefficient of thermal expansion (α) as a function of temperature for the (**a**) Si-CNT composite and (**b**) Si-CNT-Cu composite.

The dimensional stability and thermomechanical behavior of the composites were evaluated using thermomechanical analysis (TMA) over a temperature range from approximately 0 °C to 300 °C, as presented in Figure 6. The coefficient of thermal expansion (CTE, α) was determined from the linear slope of the dimension change versus temperature curve. For the bare Si-CNT membrane (Figure 6a), the dimensional change increased smoothly from 21.05 μm at −0.32 °C to 195.93 μm at 294.08 °C. The CTE calculated for this linear expansion region was 276.1 μm/m°C, which reflects the inherent thermal expansion characteristics of the carbon nanotube-reinforced silicone rubber matrix. Upon the integration of the copper wire, the Si-CNT-Cu composite (Figure 6b) exhibited a significantly more pronounced dimensional change, expanding from 49.18 μm to 307.81 μm over a similar temperature range. Consequently, the measured CTE substantially increased to 375.2 μm/m°C. This elevated thermal expansion indicates that the presence of the encapsulated Cu wire notably alters the macroscopic thermomechanical dynamics of the material. The substantial increase in the CTE can be attributed to the excellent thermal conductivity of the core copper wire, which accelerates internal heat transfer and exacerbates the thermal volume expansion of the surrounding silicone matrix. Additionally, the complex interfacial thermal stress arising from the thermomechanical mismatch between the highly elastic polymer matrix and the rigid metallic wire contributes to the amplified dimensional changes observed at elevated temperatures.

From a practical application perspective, the actual operating temperature of commercial LED modules typically ranges from room temperature to approximately 100 °C. Within this critical thermal window, minimizing the dimensional change is of paramount importance. Because this shielding membrane is designed to be directly attached to the rear printed circuit board (PCB) of the LED module, excessive thermal expansion can induce severe interfacial stress, potentially leading to warping, delamination, or detachment. Therefore, the custom-formulated Si-CNT membrane, which exhibited a significantly lower CTE (276.1 μm/m°C) and superior shape retention compared to the Cu-integrated variant, is highly advantageous. Its enhanced dimensional stability ensures reliable and conformal adhesion to the target device, confirming that the bare Si-CNT composite is the most structurally suitable membrane candidate for practical near-field noise suppression.

### 3.8. Dynamic Mechanical Analysis (DMA)

**Figure 7:**
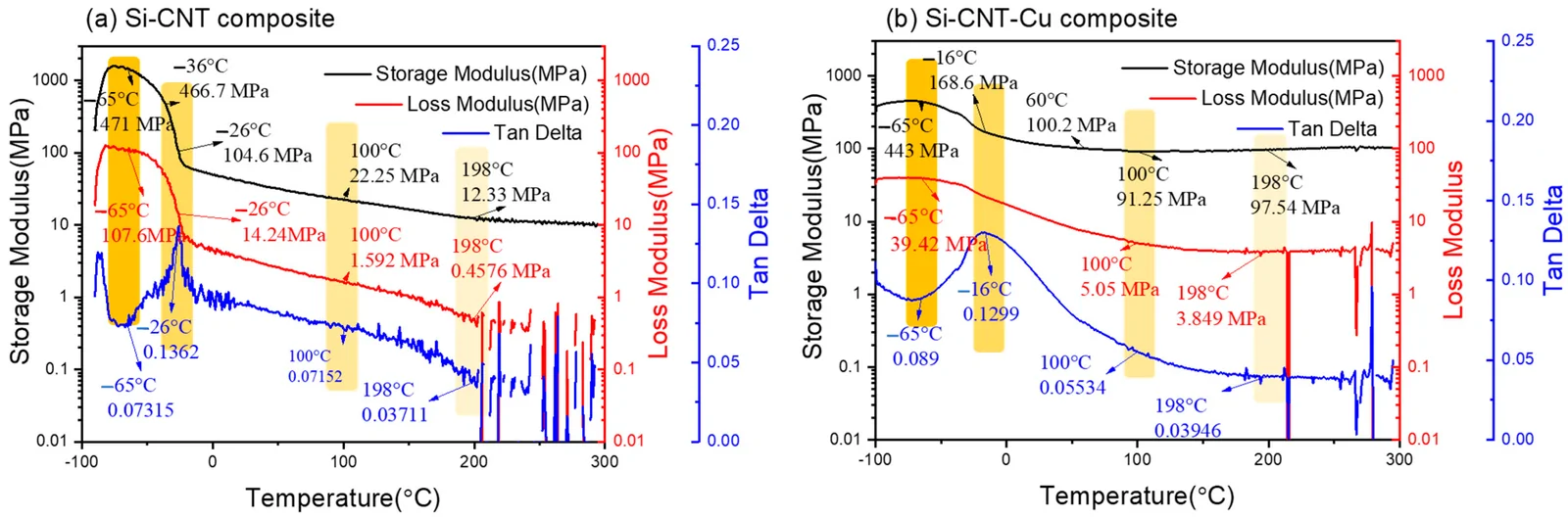
Dynamic mechanical analysis (DMA) curves illustrating the temperature dependence of storage modulus, loss modulus, and tan δ for the (**a**) Si-CNT composite and (**b**) Si-CNT-Cu composite.

The viscoelastic properties and thermomechanical stability of the composites were investigated using dynamic mechanical analysis (DMA). Figure 7 presents the temperature dependence of the storage modulus (E′), loss modulus (E″), and loss factor (tan δ) from −100 °C to 300 °C. The storage modulus curve provides critical insights into the stiffness and elastic energy retention of the materials. For the custom-formulated Si-CNT membrane (Figure 7a), the storage modulus exhibited a high value of 1471 MPa in the glassy state at −65 °C. As the temperature rises through the glass transition region, the modulus drops sharply, eventually softening to a rubbery plateau of 22.25 MPa at 100 °C and 12.33 MPa at 198 °C. In contrast, the Si-CNT-Cu composite (Figure 7b) revealed a fundamentally different thermomechanical behavior. Although its initial modulus in the deep glassy state was lower (443 MPa), it demonstrated extraordinary macro-structural stability at elevated temperatures, maintaining a high storage modulus of 91.25 MPa at 100 °C and 97.54 MPa at 198 °C. This confirms that the encapsulated rigid Cu wire acts as a robust physical reinforcement, preventing the severe high-temperature softening typically observed in bare silicone matrices. Furthermore, the tan δ curve, which represents the damping capability and is used to determine the glass transition temperature (T_g_), showed a meaningful shift. The T_g_ of the bare Si-CNT membrane was observed at approximately −26 °C. Upon the integration of the Cu wire, the tan delta (δ) peak for the Si-CNT-Cu composite shifted to a higher temperature of roughly −16 °C. This restricted segmental mobility of the polymer chains corroborates the physical constraints imposed by the rigid copper core.

When evaluating these viscoelastic properties for practical application as an LED EMI shielding membrane, the actual operating temperature of commercial LED modules (typically up to 100 °C) must be carefully considered alongside the previous TMA results. Conversely, while the Si-CNT-Cu composite offers superior structural rigidity (91.25 MPa at 100 °C), this severely compromises its conformability. The highly rigid membrane cannot flexibly adapt to the highly irregular, non-planar topography of the active driving circuits and electronic components on the PCB. This lack of conformal adhesion, combined with its high coefficient of thermal expansion (CTE), can generate severe interfacial shear stress when attached to a rigid printed circuit board (PCB). This mismatch significantly increases the risk of mechanical warping or delamination. In stark contrast, the bare Si-CNT membrane’s transition to a moderately soft rubbery state (E′ = 22.25 MPa at 100 °C) is highly advantageous for practical deployment. This lower storage modulus provides the membrane with excellent mechanical flexibility and stress-relaxation capabilities. Consequently, the Si-CNT membrane can conform perfectly to the complex topography of the LED driving circuits and effectively absorb interfacial thermomechanical shocks without detaching. Therefore, supported by both its superior dimensional stability (TMA) and optimal interfacial flexibility (DMA), the bare Si-CNT composite was definitively proven to be the more reliable and suitable shielding membrane for stable, long-term operation in commercial LED displays.

### 3.9. Correlation Between Dimensional Stability and Viscoelastic Behavior

A comprehensive understanding of the composite materials’ high-temperature performance and structural reliability can be achieved by correlating the unconstrained dimensional changes observed in TMA (Figure 6) with the dynamic viscoelastic properties evaluated in DMA (Figure 7). For the bare Si-CNT membrane, TMA results indicated a relatively lower dimensional change with a CTE of 276.1 μm/m°C (Figure 6a). However, the DMA results revealed a structural softening at elevated temperatures, where the storage modulus dropped to 22.25 MPa at 100 °C and further to 12.33 MPa at 198 °C (Figure 7a). This demonstrates that while the intrinsic silicone–CNT network exhibits a lower inherent volume expansion upon heating, it transitions fully into a flexible rubbery state at typical operating temperatures, lacking high macro-structural rigidity under extreme mechanical loads.

Conversely, the Si-CNT-Cu composite exhibited a higher dimensional expansion in TMA (CTE of 375.2 μm/m°C, Figure 7a) but maintained a remarkably high and stable storage modulus of 91.25 MPa at 100 °C and 97.54 MPa at 198 °C in DMA (Figure 7b). This apparent contrast highlights the dual role of the encapsulated Cu wire depending on the applied physical conditions. Under the stress-free heating condition of TMA, the excellent thermal conductivity of the copper core rapidly transfers heat to the internal matrix, and the thermal expansion mismatch between the rigid metal and the highly elastic polymer exacerbates the overall macroscopic dimensional change. However, under the dynamic mechanical loading conditions of DMA, the rigid Cu wire functions as a robust macro-structural reinforcement. It effectively absorbs and dissipates the applied mechanical stress, preventing the severe macroscopic deformation and thermal softening that typically occur in bare silicone matrices. Ultimately, while the Cu wire accelerates the internal thermal expansion volume, it simultaneously provides a crucial load-bearing framework required to maintain the mechanical integrity of the composite at extreme temperatures. Concurrently, its moderate viscoelastic softening (22.25 MPa at 100 °C) allows the flexible membrane to undergo effective stress relaxation. Although there is a large CTE mismatch between the Si-CNT membrane (276.1 μm/m°C) and standard rigid FR-4 PCB substrates (~14–17 μm/m°C), the extremely low elastic modulus of the rubbery silicone matrix acts as a compliant buffer. It absorbs the thermal expansion strain through elastic deformation rather than building up severe interfacial shear stress, thereby preventing delamination and maintaining conformal contact with the complex, non-planar topography of the rear driving circuits.

From an engineering standpoint for practical LED display applications, correlating these two distinct thermomechanical profiles reveals that the bare Si-CNT membrane serves as the optimized selection for direct device attachment. Within the standard operating window of commercial LED modules (up to 100 °C), the Si-CNT membrane guarantees superior dimensional compliance due to its lower CTE, which prevents excessive volume expansion. Concurrently, its moderate viscoelastic softening (22.25 MPa at 100 °C) allows the flexible membrane to undergo effective stress relaxation and maintain conformal contact with the complex, non-planar topography of the rear driving circuits. In contrast, although the Si-CNT-Cu product offers outstanding rigid load-bearing capabilities, its combination of high thermal expansion mismatch and extreme high-temperature stiffness can generate significant interfacial shear stress against a rigid printed circuit board (PCB). This thermomechanical conflict markedly elevates the risk of interfacial warping, cracking, or delamination during prolonged thermal cycling. Consequently, while the Cu-integrated framework excels in standalone structural scenarios, the custom-formulated Si-CNT membrane was definitively proven to be the more reliable, compatible, and practically viable candidate for long-term localized near-field EMI shielding applications.

### 3.10. Electromagnetic Interference (EMI) Shielding Performance and Mechanism

The electromagnetic interference (EMI) shielding performance of the fabricated materials was evaluated by measuring the scattering parameters (S-parameters) in the frequency range of 0.4 to 1.6 GHz. When the surface of a shielding material is exposed to incident electromagnetic (EM) radiation, the wave energy is initially attenuated by reflection due to the impedance mismatch between the shielding medium and free space. The non-reflected portion that penetrates the material is subsequently dissipated through internal absorption. Furthermore, when the un-reflected waves reach the internal boundaries, they can undergo multiple internal reflections that continuously contribute to the overall attenuation of the EM energy [16]. To fundamentally elucidate the shielding mechanism, the total shielding effectiveness (SE_T_) can be decoupled into reflection shielding effectiveness (SE_R_) and absorption shielding effectiveness (SE_A_), and multiple reflection (SE_m_) components based on Schelkunoff’s theorem and the measured scattering coefficients (S_11_ and S_21_) [16,42]. The relative power fractions—reflectivity (R), transmissivity (T), and absorptivity (A)—and their corresponding SE components are quantitatively determined by the following mathematical relationships:

R = |S_11_|^2^, T = |S_21_|^2^, A + R + T = 1

SE_r_ = −10 log(1 − R), SE_a_ = −10 log(T/(1 − R))

SE_t_ = SE_r_ + SE_a_ + SE_m_

Generally, the multiple reflection term (SE_m_) can be safely disregarded in the event that the total SE is over 10 dB. Therefore, the overall relationship for the highly effective shielding materials in this study is practically defined as SE_t_ ≈ SE_r_ + SE_a_ = −10 log(T) [16].

**Figure 8:**
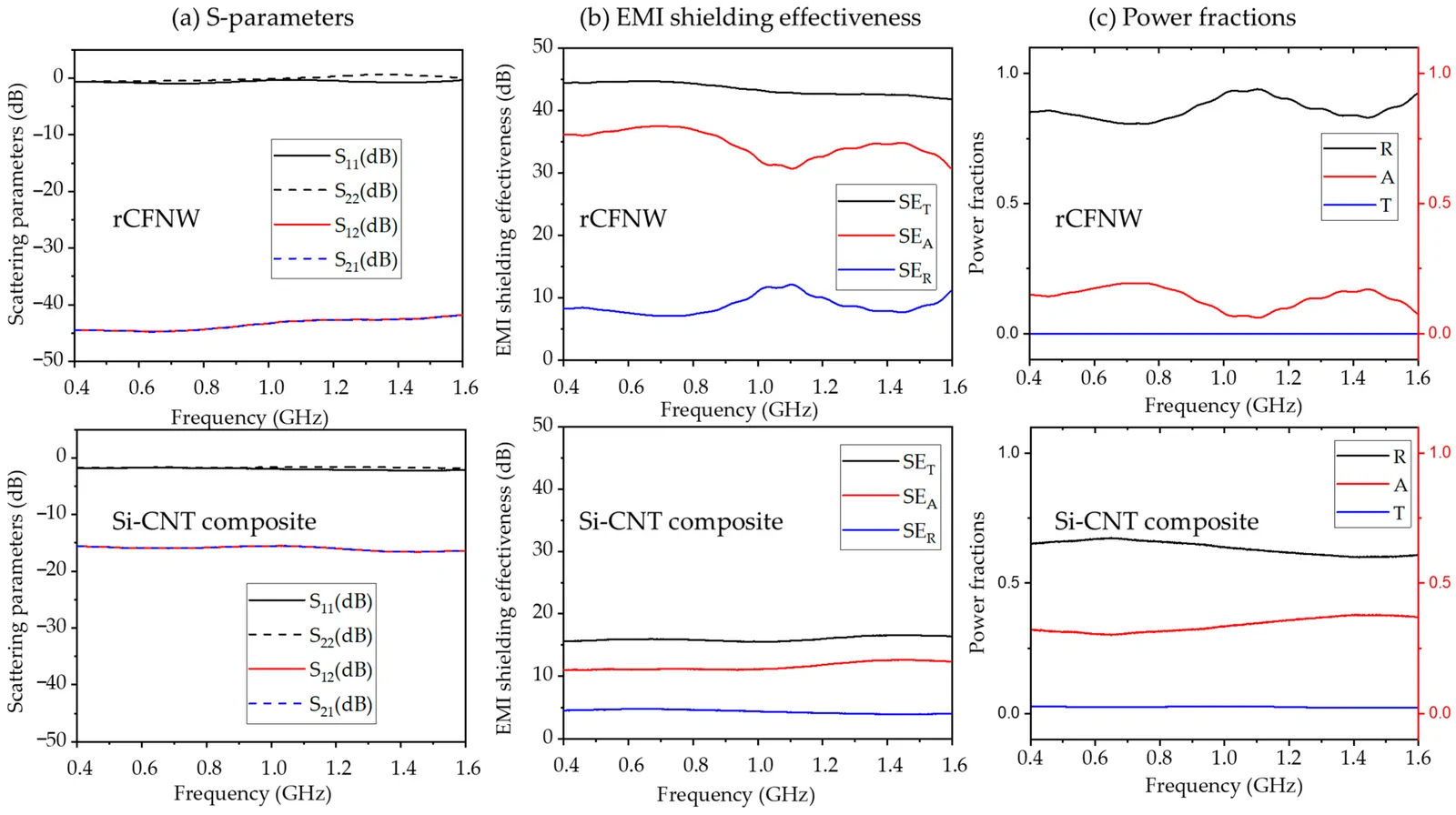
Comparative analysis of the electromagnetic interference (EMI) shielding properties for the pristine rCFNW (**top row**) and the Si-CNT composite (**bottom row**) in the 0.4–1.6 GHz frequency range. (**a**) Measured scattering parameters (S_11_, S_22_, S_12_, and S_21_). (**b**) EMI shielding effectiveness, showing the total shielding (SE_t_) decoupled into absorption (SE_a_) and reflection (SE_r_) components. (**c**) Calculated power fractions of the incident electromagnetic waves, demonstrating the transition in reflectivity (R), absorptivity (A), and transmissivity (T).

Figure 8 illustrates the EMI shielding mechanisms for the pristine recycled carbon fiber nonwoven (rCFNW) in the top row and the custom-formulated Si-CNT membrane in the bottom row. For the pristine rCFNW (Figure 8a, top), the reflection coefficients (S_11_ and S_22_) fluctuated very close to 0 dB (ranging from roughly −0.5 dB to 1.0 dB). Values approaching 0 dB indicate a strong initial reflection of the incident electromagnetic waves. As clarified by the power fraction analysis in Figure 8c (top), the abundant free electrons on the surface of the highly conductive carbon fiber network interact vigorously with the incoming radiation, reflecting a substantial portion of it immediately upon contact (R > 0.8). Consequently, the transmission coefficients (S_12_ and S_21_) in Figure 8a were consistently maintained at extremely low levels, strictly between −42.0 dB and −44.0 dB. This translates to an outstanding SE_t_ of approximately 43 dB, as shown in Figure 8b. The remarkable value of ~43 dB achieved by the bare rCFNW indicates that it effectively blocks over 99.99% of the incident electromagnetic energy, verifying that the highly interconnected graphitic structure acts as a robust intrinsic conductive framework.

Following the evaluation of the pristine carbon network, the EMI shielding performance of the custom-formulated Si-CNT membrane was analyzed in the same frequency range. The integration of the carbon nanotubes and the recycled carbon fibers into the silicone rubber matrix induces a fundamental shift in the electromagnetic wave attenuation behavior. As shown in Figure 8a (bottom), the reflection coefficients dropped to more negative values, ranging from approximately −1.5 dB to −2.5 dB. As quantitatively proven in Figure 8c (bottom), this reduction occurs because the silicone matrix acts as an impedance-matching dielectric medium at the composite interface, significantly lowering the power reflectivity (R) and allowing a larger portion of the microwaves to penetrate the surface rather than bouncing off instantly. As a result of this polymer encapsulation, the transmission coefficients (S_12_ and S_21_) fluctuated between −15.5 dB and −16.5 dB, which translates to an SE_t_ of approximately 16 dB (Figure 8b). While this represents a quantitative reduction compared to the ~43 dB of the pristine rCFNW—due to the silicone matrix partially insulating the continuous electron transport pathways—the highly elastic membrane still effectively blocked over 97% of the incident electromagnetic waves. More importantly, this intentional reduction in surface reflection simultaneously promotes internal absorptivity (A). As explicitly demonstrated in the power fractions (Figure 8c), the incident microwaves that penetrate the surface become trapped within the composite, undergoing multiple internal reflections between the embedded CNTs and the separated carbon fibers until the electromagnetic energy is dissipated as heat. This enhanced absorption-driven phenomenon is highly consistent with previous findings, which demonstrated that hybridizing macroscopic carbon fabrics with nanoscale carbon fillers dramatically improves the internal scattering and wave shielding effectiveness [43]. To support this absorptive behavior, the surface electrical resistance (sheet resistance) of the 3 wt% Si-CNT composite membrane was quantitatively evaluated. Unlike the purely insulating bare silicone matrix, the 3 wt% composite exhibited a highly stable surface resistance of 27.89 ± 2.01 Ω/cm^2^. This low sheet resistance clearly confirms that the composite operates well above the electrical percolation threshold, establishing a robust internal 3D conductive network necessary for the efficient ohmic dissipation of the penetrating electromagnetic waves without inducing excessive instantaneous surface reflection.

It is crucial to note that while the silicone matrix acts as a dielectric spacer for electromagnetic absorption, the uniformly dispersed CNTs and carbon fibers impart a partial macroscopic electrical conductivity to the overall Si-CNT membrane. Consequently, applying this shielding membrane directly onto the bare printed circuit board (PCB) of the LED module could induce severe electrical short circuits or operational shocks. To prevent this, in the subsequent practical near-field evaluations, the rear driving circuits of the LED module were first treated with a conformal insulating layer before attaching the Si-CNT shielding membrane. This critical integration strategy ensures both operational electrical stability and highly effective, absorption-dominant near-field noise suppression.

### 3.11. Practical Near-Field Application Setup and Baseline EMI Evaluation

**Figure 9:**
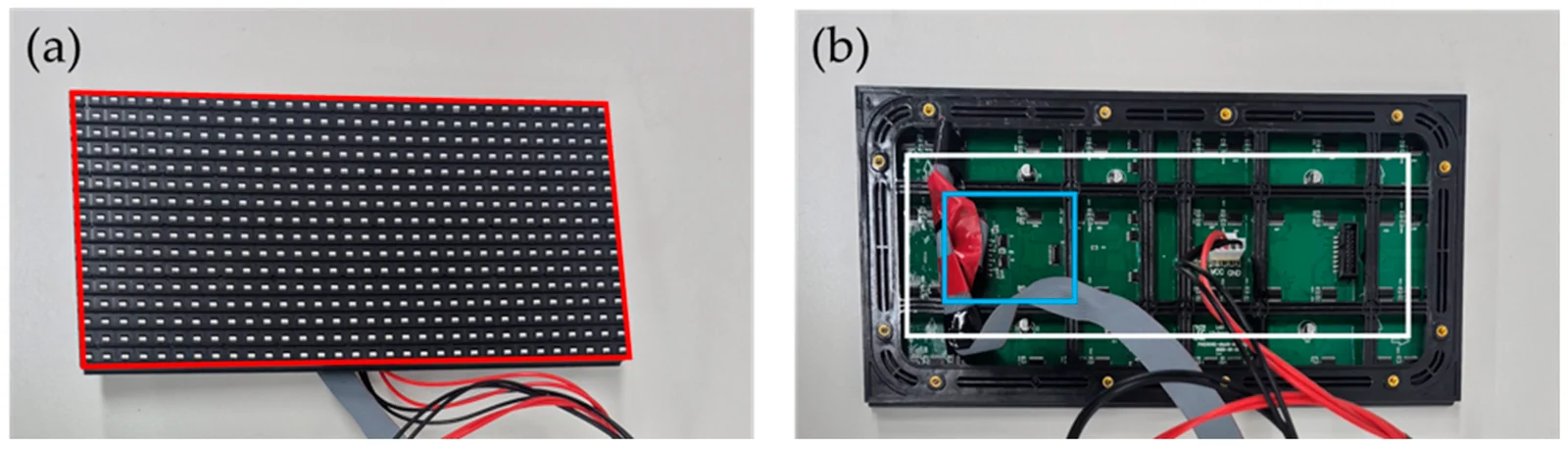
Experimental setup for the baseline electromagnetic interference (EMI) measurements of the bare LED display system without shielding material: (**a**) front side of the LED module, and (**b**) rear side of the LED module exposing the driving circuits.

To evaluate the practical electromagnetic interference (EMI) shielding capability of the composite materials in a real-world electronic device, a commercial LED dot matrix display module was utilized as the primary electromagnetic noise source. Before evaluating the shielding effectiveness, it is essential to establish an accurate reference point by measuring the intrinsic electromagnetic radiation emitted from the bare device without any shielding material. As illustrated in Figure 9, the baseline near-field EMI measurements were systematically conducted on distinct radiative regions of the LED setup. Specifically, the red rectangle in Figure 9a indicates the precise measurement area on the front face of the module, primarily consisting of the diode arrays. Conversely, Figure 9b exposes the rear side of the module containing the printed circuit board (PCB) and high-frequency driving ICs. Here, the white rectangle outlines the broad baseline measurement area. Furthermore, the blue rectangle denotes the specific localized target area over the main driving circuits where the shielding membrane was subsequently applied for comprehensive shielding performance analysis.

**Figure 10:**
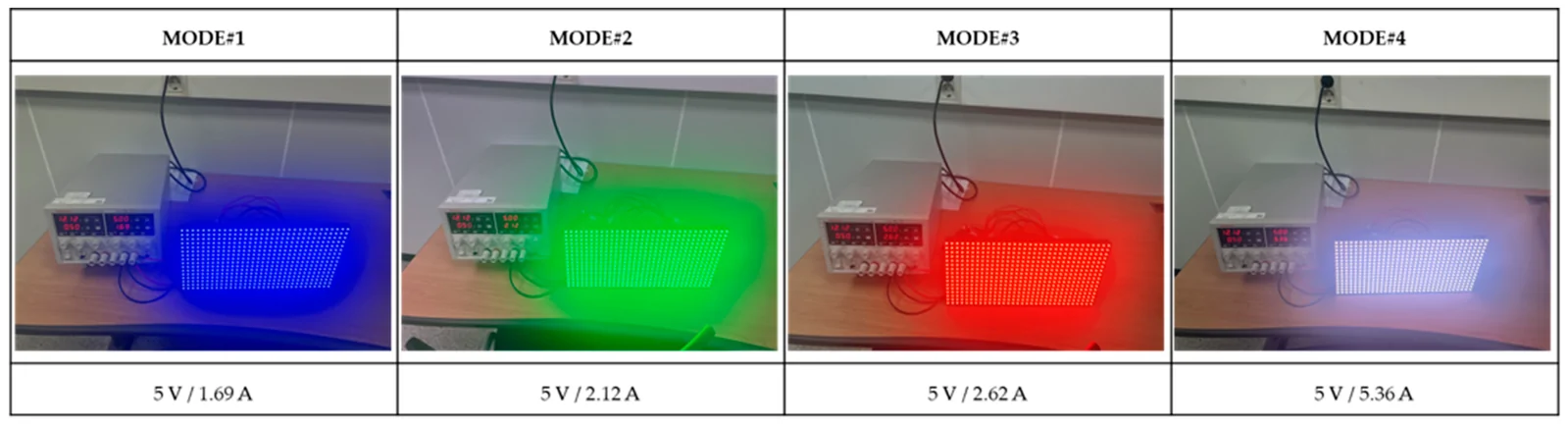
Power consumption analysis of the commercial LED dot matrix module under different full-screen operating modes at a constant 5 V supply: blue (Mode #1), green (Mode #2), red (Mode #3), and white (Mode #4). Mode #4 was selected for the EMI shielding test due to its highest current draw (5.36 A), representing the worst-case electromagnetic radiation scenario.

The intensity of radiated electromagnetic noise from these active electronic components is directly proportional to their operating current. Therefore, preliminary measurements were conducted to determine the power consumption across four different full-screen display modes under a constant supply voltage of 5 V, as presented in Figure 10. The measured current consumption varied depending on the operating color: 1.69 A for the blue light (Mode #1), 2.12 A for the green light (Mode #2), and 2.62 A for the red light (Mode #3). Notably, the white light emission (Mode #4) requires the simultaneous activation of all internal red, green, and blue (RGB) diodes, resulting in the maximum current consumption of 5.36 A.

According to fundamental electromagnetic principles, a higher operating current induces stronger surrounding magnetic fields and subsequent electromagnetic radiation. Therefore, Mode #4 represents the most severe electromagnetic noise environment among the available operations. By recording the radiated noise from the bare components under this maximum power consumption condition (white light, 5.36 A), an accurate unshielded reference profile was obtained. The front surface typically emits lower-level noise from the light-emitting components, whereas the rear surface acts as a severe broadband noise source due to the dense switching circuits and digital signal transmission. Establishing this bare-state radiation profile under the worst-case scenario is a critical methodological step, as it allows for the precise quantification of the net EMI shielding performance once the engineered shielding materials are applied over these specific regions.

### 3.12. Spatial and Spectral EMI Analysis of the Bare LED System

**Figure 11:**
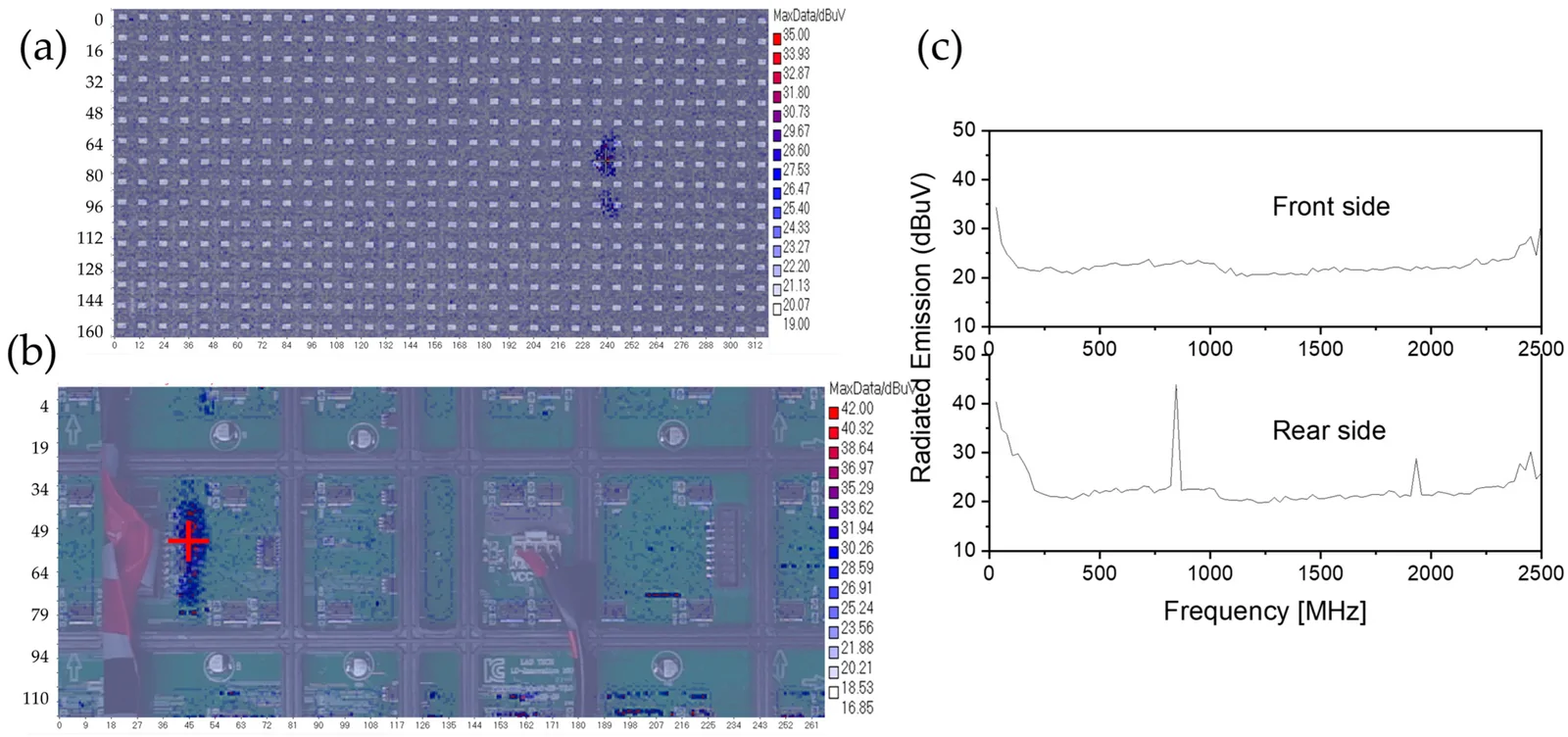
Baseline electromagnetic interference (EMI) characteristics of the bare LED module: 2D spatial EMI mapping of the (**a**) front side and (**b**) rear side, and (**c**) the corresponding electromagnetic radiation frequency spectra (0 to 2500 MHz) for both sides.

To quantitatively assess the intrinsic electromagnetic radiation characteristics before applying the shielding material, the spatial distribution and frequency spectra of the bare LED module were analyzed, as presented in Figure 11. The measurements were conducted under the maximum power consumption condition (white light emission) to capture the worst-case radiation scenario.

The 2D spatial EMI mappings were systematically acquired by scanning the specific target regions outlined in the previous setup: the red rectangular area (Figure 9a) on the front side and the white rectangular area on the rear side. As shown in Figure 11a, the scanned region on the front side exhibited a relatively uniform and low-intensity electromagnetic field, with most areas showing minimal radiation levels. This is theoretically sound, as the front surface consists purely of light-emitting diodes that generate visible photons rather than severe radio-frequency waves. Conversely, the spatial mapping of the scanned white rectangular area (Figure 9b) on the rear side (Figure 11b) revealed intense, localized electromagnetic hotspots, with the maximum radiation intensity reaching up to approximately 42 dBuV. Notably, the exact location of this peak emission is marked by a red crosshair (+) in Figure 11b. These high-intensity areas perfectly align with the physical locations of the high-frequency driving integrated circuits (ICs) and digital signal routing pathways on the exposed printed circuit board.

The spectral analysis in the frequency range of 0 to 2500 MHz, illustrated in Figure 11c, further corroborates the spatial observations. The electromagnetic spectrum of the front side remained relatively flat and stable, hovering around 20 dBuV across the entire measured band. In stark contrast, the spectrum extracted specifically from the red crosshair (+) location on the rear side exhibited significantly higher noise levels characterized by sharp, prominent peaks. Most notably, a severe radiation peak exceeding 40 dBuV was observed at approximately 850 MHz, along with another distinct peak near 1950 MHz. These strong, narrow-band spikes are typical signatures of the fast-switching clock signals and data transmission rates generated by the display controllers.

**Figure 12:**
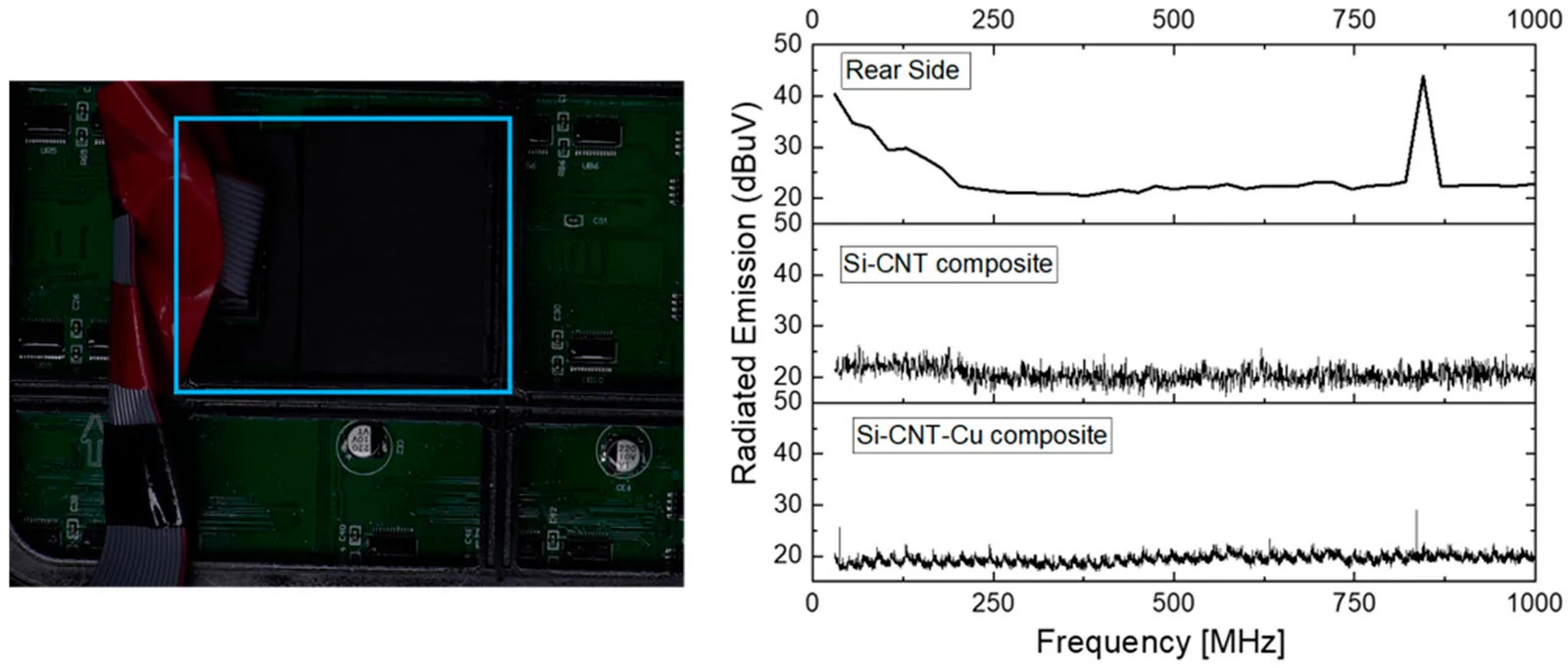
Practical EMI shielding application results: (**left**) photograph showing the composite material applied over the targeted electromagnetic hotspot (blue rectangle) on the rear side of the LED module, and (**right**) comparative radiated emission spectra (0–1000 MHz) demonstrating the complete suppression of the severe 850 MHz noise peak by both the Si-CNT and Si-CNT-Cu composites.

Collectively, these baseline results explicitly demonstrate that the rear circuitry is the primary source of severe electromagnetic leakage in the display system. Consequently, these unshielded measurements establish a precise reference profile, confirming that the application of the Si-CNT-Cu composite should be strategically targeted at the rear side to effectively suppress these high-frequency switching noises. Collectively, these baseline results explicitly demonstrate that the rear circuitry is the primary source of severe electromagnetic leakage in the display system. Consequently, these unshielded measurements establish a precise reference profile. Specifically, the severe emission recorded at the crosshair (+) serves as the exact baseline for the subsequent shielding evaluation in Figure 12, confirming that the application of the engineered shielding materials should be strategically targeted at this localized rear circuitry (the blue rectangular area in Figure 9b) to effectively suppress these high-frequency switching noises.

### 3.13. Practical EMI Shielding Application of the Composites

To validate the practical shielding effectiveness of the developed materials in a real-world electronic environment, the custom-formulated shielding composite materials were directly applied over the previously identified electromagnetic hotspot on the rear circuitry of the LED module. Prior to application, a conformal insulating layer was carefully applied to the exposed printed circuit board (PCB) to prevent any electrical short circuits from the partially conductive carbon networks. As shown in the left panel of Figure 12, the shielding membrane was strategically attached to cover the high-frequency driving ICs, precisely corresponding to the blue rectangular target area previously defined in Figure 9b. The radiated emission was then measured under the same maximum power consumption condition (white light emission) to evaluate the localized noise suppression capability. The right panel of Figure 12 presents a direct comparison of the radiated emission spectra in the frequency range of 0 to 1000 MHz before and after the application of the shielding materials. The top spectrum, representing the unshielded baseline extracted from the exact crosshair (+) location in Figure 11b, clearly exhibited a severe, narrow-band radiation spike exceeding 40 dBuV at approximately 850 MHz. This intense peak acted as the primary target for the shielding test. Upon covering the hotspot with the Si-CNT membrane (middle spectrum), the prominent 850 MHz peak was completely eradicated. The radiated emission level dropped drastically, flattening out to merge with the background noise floor at approximately 20 to 25 dBuV across the entire frequency band. Furthermore, the application of the Si-CNT-Cu composite (bottom spectrum) demonstrated an equally exceptional, highly stable noise suppression performance. The severe high-frequency switching noise was thoroughly neutralized without any secondary radiation leakage. This dramatic reduction in near-field radiated emission highlights the most critical finding of this study. It unequivocally proves that overcoming the reflective inefficiency inherent to highly conductive pure carbon networks through absorptive suppression is a highly effective strategy. While the incorporation of the insulating silicone matrix quantitatively lowers the theoretical far-field shielding effectiveness compared to the bare rCFNW, the resulting Si-CNT and Si-CNT-Cu composite materials avoid severe secondary wave reflections by predominantly absorbing the trapped near-field noise. Consequently, these flexible, absorption-driven composites are proven to be highly capable of functioning as practical, localized EMI shielding barriers, effectively securing commercial electronic devices against severe electromagnetic interference.

### 3.14. EMI Shielding Mechanism: Near-Field vs. Far-Field Considerations

The apparent discrepancy between the theoretical far-field shielding effectiveness measured by the VNA and the practical near-field noise suppression observed in the active LED module highlights the critical importance of the underlying shielding mechanism. As previously demonstrated in the far-field VNA results (Figure 8), the bare rCFNW exhibited an outstanding transmission suppression (S_21_ of approximately −43 dB). However, its reflection coefficients (S_11_ and S_22_) hovered exceptionally close to 0 dB, indicating that the pristine carbon network acts essentially as an electromagnetic mirror.

When applied directly over noise-emitting high-frequency ICs in a localized near-field environment, this purely reflection-dominant mechanism induced severe reflective inefficiency. Instead of eliminating the electromagnetic energy, the highly reflective rCFNW caused the radiated waves to bounce back and forth between the shield and the underlying circuitry. This secondary wave reflection generates severe signal interference within the driving electronic components and allows the trapped noise to leak through microscopic physical gaps, ultimately rendering the high far-field shielding value practically meaningless.

Conversely, the custom-formulated Si-CNT and Si-CNT-Cu composite materials, despite showing a theoretically lower far-field transmission suppression (S_21_ of approximately −16 dB) in Figure 8, achieved absolute near-field noise eradication in the practical LED evaluation. This superior real-world performance is attributed to a fundamental transition in the shielding mechanism—moving away from surface reflection toward internal absorptive suppression. The incorporation of the silicone matrix inherently modifies the surface properties of the material, optimizing the interfacial impedance. Consequently, the reflection coefficients (S_11_ and S_22_) dropped to more negative values, ranging from −1.5 dB to −2.5 dB. As quantitatively confirmed by the power fraction analysis, this intentional decrease in surface reflectivity establishes a crucial impedance-matching interface, allowing the incident near-field electromagnetic waves to penetrate the membrane rather than bouncing off instantly, thereby dissipating the noise effectively through enhanced internal absorptivity (A).

Once the electromagnetic waves enter the membrane layer, they become trapped within the highly complex three-dimensional conductive network formed by the encapsulated CNTs and the embedded carbon fibers. The incident energy undergoes multiple internal reflections between these micro- and nano-scale conductive fillers and is seamlessly dissipated as heat via ohmic losses and polarization relaxation within the dielectric silicone matrix. The long-term structural reliability and robust thermal stability of such carbon fiber nonwoven fabrics under continuous heating conditions have been thoroughly validated in our previous investigations [16,44], ensuring that the composite can safely withstand the thermal stress generated during this electromagnetic-to-thermal energy conversion.

Therefore, while the silicone matrix quantitatively reduces the absolute far-field VNA shielding value by disrupting continuous electron pathways, it critically converts the composite into an absorption-dominant shielding barrier. Ultimately, this absorption-driven mechanism completely suppresses the severe 850 MHz switching noise without inducing secondary reflections. Coupled with the tailored thermomechanical profiles established in the previous sections—where the bare Si-CNT membrane offers optimized dimensional stability for conformal device attachment and the Si-CNT-Cu variant provides robust load-bearing framework—this study provides a comprehensive design strategy for reliable, high-performance near-field EMI shielding in high-power, densely integrated commercial electronics. In this study, to clearly demonstrate the fundamental paradigm shift from the purely reflective bare rCFNW to an absorption-dominant mechanism, the exact CNT loading in the evaluated composites was fixed at 3 wt%. A comprehensive and systematic investigation detailing the effects of varying CNT concentrations (3–10 wt%) on the percolation threshold and shielding mechanisms will be reported in a subsequent future publication.

## 4. Conclusions

This study conducted a comprehensive evaluation of a high-performance custom-formulated Si-CNT composite and its Cu-integrated variation (Si-CNT-Cu) as localized EMI shielding composite materials for commercial LED displays. Through morphological, chemical, and thermal investigations using FE-SEM, Raman spectroscopy, XPS, ICP-AES, FTIR, and TGA-DTG, the successful manufacturing and strong structural integration of the carbon network, silicone matrix, and macroscopic Cu wire were thoroughly verified. Importantly, these analyses confirmed that the homogeneous integration of 3 wt% carbon nanotubes (CNTs) into the naturally non-conductive silicone matrix effectively established an interconnected conductive network (27.89 Ω/cm^2^), providing the necessary electrical characteristics for efficient EMI shielding.

Most importantly, our study addressed the apparent disparity between near-field practical application and far-field theoretical shielding. Although the pure rCFNW demonstrated a better far-field EMI SE_T_ of around 43 dB, its near-zero reflection coefficients induced extreme power reflectivity (R > 0.8) and strong secondary wave reflection, making it extremely inefficient in localized near-field situations. By utilizing the silicone matrix to optimize the interfacial impedance, the custom-formulated Si-CNT composite was successfully transformed into a highly effective, absorption-dominant shielding membrane, a mechanism quantitatively verified by power fraction analysis (R, T, A). Consequently, during practical near-field evaluations rigorously validated at an official testing institute (JIAT), the Si-CNT membrane effectively eliminated the severe 850 MHz switching noise peak (exceeding 40 dBuV) on the driving circuitry of the LED module working at a maximum 5.36 A, seamlessly reducing it to the background noise level. This confirms that the Si-CNT composite can be preferentially utilized as an excellent primary near-field noise suppression barrier.

Additionally, in the thermomechanical analysis (TMA), the Si-CNT membrane possessed a lower coefficient of thermal expansion (CTE) compared to the Si-CNT-Cu variant. Furthermore, the crucial significance of the encapsulated Cu wire in the Si-CNT-Cu version for high-temperature applications was clearly shown by dynamic mechanical analysis (DMA). The Si-CNT-Cu composite successfully overcame the severe thermal softening typically seen in bare silicone matrices by maintaining an extraordinarily stable storage modulus of 97.54 MPa at high temperatures (198 °C). In contrast, the optimally flexible Si-CNT membrane avoids generating severe interfacial shear stress, making it the definitive choice for conformal PCB attachment. In the end, these results clearly demonstrate that while the Cu-reinforced structure offers an excellent structural framework, the engineered bare Si-CNT shielding composite provides a highly dependable, practically feasible, and reliable solution for mitigating severe near-field electromagnetic interference in high-power commercial electronics. While the present study successfully demonstrates the fundamental electromagnetic shielding mechanisms and practical near-field noise suppression of the flexible Si-CNT membranes, further comprehensive evaluations regarding the effects of varying CNT concentrations (3–10 wt%), alongside long-term environmental reliability tests—such as mechanical tensile and peel-strength retention under high-humidity and thermal-shock conditions—are currently underway to fully establish their robust industrial viability for next-generation electronic systems.

## Figures and Tables

**Table 1:** Elemental composition (atomic %) of the samples: (a) rCFNW, (b) Si-CNT composite, and (c) Si-CNT-Cu composite.

Elements	(a) rCFNW	(b) Si-CNT Composite	(c) Si-CNT-Cu Composite
C	91.7	43.81	44.3
O	2.56	35.69	32.87
N	5.74	none	n.d.
Si	^1^ n.d.	20.3	19.04
Others	n.d.	0.2	3.79

^1^ n.d. indicates that the element was not detected. All values are expressed in atomic percentage (atomic %).

## Data Availability

The original contributions presented in this study are included in the article. Further inquiries can be directed to the corresponding author.
